# Review of Quantitative Microbial Risk Assessment in Poultry Meat: The Central Position of Consumer Behavior

**DOI:** 10.3390/foods9111661

**Published:** 2020-11-13

**Authors:** Tahreem Khalid, Ammar Hdaifeh, Michel Federighi, Enda Cummins, Géraldine Boué, Sandrine Guillou, Vincent Tesson

**Affiliations:** 1 SECALIM, INRAE, Oniris, 44307 Nantes, France; tahreem.khalid@oniris-nantes.fr (T.K.); ammar.hdaifeh@oniris-nantes.fr (A.H.); michel.federighi@oniris-nantes.fr (M.F.); geraldine.boue@oniris-nantes.fr (G.B.); vincent.tesson@inrae.fr (V.T.); 2 Biosystems and Food Engineering, University College Dublin, Belfield, Dublin 4, Ireland; enda.cummins@ucd.ie

**Keywords:** food safety, QMRA, meat chain, poultry, food preparation, consumer behavior

## Abstract

Food of animal origin, especially meat products, represent the main vehicle of foodborne pathogens and so are implicated in foodborne outbreaks. Poultry meat is a widely consumed food in various forms, but it is also a reservoir of thermotolerant *Campylobacter* and *Salmonella* bacterial species. To assess human health risks associated with pathogenic bacteria in poultry meat, the use of quantitative microbial risk assessment (QMRA) has increased over the years as it is recognized to address complex food safety issues and is recommended by health authorities. The present project reviewed poultry meat QMRA, identified key steps of the farm-to-fork chain with significant impacts on food safety, highlighted current knowledge gaps, and provided risk mitigation advices. A PRISMA (Preferred Reporting Items for Systematic Reviews and Meta-Analyses)-based systematic analysis was carried out and enabled the collection of 4056 studies including 43 QMRA kept for analysis after screening. The latter emphasized *Campylobacter* spp. and *Salmonella* spp. contaminations during the consumer stage as the main concern. The role of consumer handling on cross-contamination and undercooking events were of major concern. Thus, proper hygiene and safety practices by consumers have been suggested as the main intervention and would need to be followed with regular surveys to assess behavior changes and reduce knowledge gaps.

## 1. Introduction

According to the World Health Organization (WHO) in 2010, around 600 million cases of foodborne illness and 420,000 deaths were reported due to the consumption of food contaminated by enteric pathogens worldwide [1]. In the EU, there were around 5146 foodborne outbreaks in 2018, with meat and related products a major source of infection, responsible for 17.9% of strong-evidence foodborne outbreaks in 2018 [2]. The most common agent detected was *Salmonella* spp. with *Salmonella* ser. Enteritidis, which caused one out of three outbreaks. It was followed by bacterial toxins then *Campylobacter* spp. (12.7% and 10.2% of reported outbreaks, respectively) [2]. *Salmonella* spp. in eggs and meat and meat products were the top ranked hazard/food pairs [2]. The presence of foodborne microbial pathogens along the poultry meat chain is one of the major public health concerns. Poultry is often known as a reservoir of human enteric pathogens such as thermotolerant *Campylobacter* spp. and *Salmonella* spp., mostly present in the intestinal tract of birds, which often appear asymptomatic [3]. In 2017, the EU flock prevalence of target *Salmonella* spp. serovars in breeding hens, laying hens, broilers, and fattening turkeys slightly decreased or remained stable compared to 2017 [2]. In a report of European Center for Diseases Control (ECDC) and European Food Safety Authority (EFSA), campylobacteriosis was shown to be the most commonly reported zoonosis and the EU trend for confirmed human cases has increased since 2008 and remained stable during 2014–2018 [2]. Human campylobacteriosis is most commonly associated with the consumption of poultry, specifically fresh, portioned, or whole broiler meat products [4,5,6]. Moreover, broiler is highly consumed worldwide, estimated at 13.9 kg per capita in 2015–2017 and expected to increase up to 14.6 kg per capita up to 2027 [7]. In the EU from 2015 to 2017, the average poultry meat consumption was higher with 23.74 kg per capita and an increase up to 24.9 kg per capita is expected by 2027 [7]. In comparison, the French are large consumers with approximately 26.3 kg per capita of (90% broiler meat) consumed in 2014 [8]. Poultry meat is purchased in the form of whole carcasses, cutting parts, and other elaborated products, which accounts for 25%, 44%, and 31%, respectively [8].

To assess the risk associated with poultry consumption and evaluate risk reduction measures, Quantitative Microbial Risk Assessment (QMRA) is used as a structured approach that enables the estimation of the probability of illness to which people may be exposed. It consists of determining the level of contamination by human/animal related pathogens, which may represent a risk to human health [9]. QMRA consists of four steps: hazard identification, hazard characterization, exposure assessment, and risk characterization [10]. The poultry meat chain comprises several steps starting from the breeder to the consumption. There are several possible routes of contamination along the farm-to-fork chain of poultry meat products, from the colonization of the chick gut by microbial pathogens at the farm level to their spread and growth during the slaughter process, the retail and preparation steps, and till consumption. Thus, QMRA can be useful in providing a food safety approach to minimize the risk of pathogen exposure and potential resulting health issues.

In the present paper, a critical analysis of existing QMRA studies related to poultry meat products in the farm-to-fork chain was performed. The objective of this analysis was to pinpoint knowledge gaps and issues within the critical steps of the poultry chain, especially the consumer step, to identify how an improved QMRA may refine intervention strategies.

## 2. Materials and Methods

The scientific papers were collected through exhaustive literature search following the PRISMA guidelines [11,12] on Web of Science (WoS) and Scopus. For each database, the following search queries were used:

- WoS: TITLE: (poultry OR chicken* OR broiler* OR poulet* OR volaille* OR duck* OR geese* OR turkey* OR dinde* OR oie* OR canard*) AND TITLE: (risk* OR risque* OR “risk assessment” OR aqr OR QMRA OR exposure OR “modelling”)
- Scopus: (TITLE (poultry OR chicken* OR broiler* OR poulet* OR volaille* OR duck* OR geese* OR turkey* OR dinde* OR oie* OR canard*) AND TITLE (risk* OR risque* OR “risk assessment” OR aqr OR qmra OR exposure OR modeling OR modelling).

The search was done on each database considering their respective starting year—1956 for Web of Science, 1788 for Scopus—to the date of the 11 February 2020. A total of 4565 articles (2242 from Web of Science and 2323 from Scopus) were collected, and 32 papers from the grey literature (mostly reports from European health agencies and the WHO) were added. Then, articles were screened that involved the removal of duplicates and scrutiny of titles and abstracts. Records were considered eligible after thorough inspection of full texts. During the whole selection process, only articles that met potential inclusion criteria were included: studies related to poultry QMRA, predictive modeling, risk factors, and prevalence of poultry pathogens. The studies were included without geographical discrimination. Studies that did not comply with the inclusion criteria were rejected.

## 3. Results

### 3.1. Summary of Collected Papers

**Figure 1:**
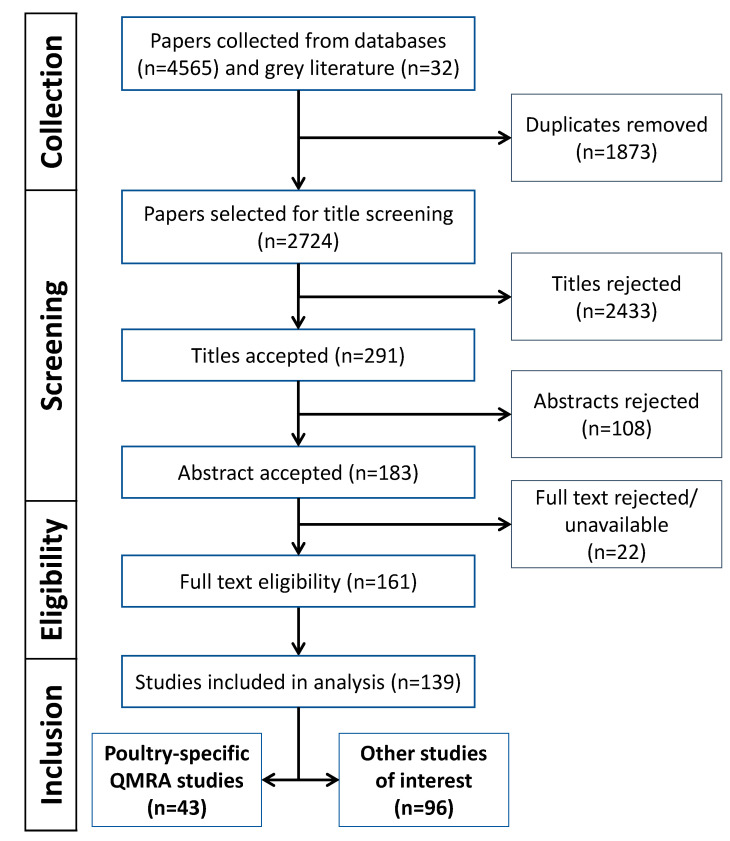
Flow chart of the data search process and results, based on the PRISMA diagram [11,12].

A total of 139 studies were collected after screening through the PRISMA method in which 43 studies specifically concerned poultry QMRA [8,13,14,15,16,17,18,19,20,21,22,23,24,25,26,27,28,29,30,31,32,33,34,35,36,37,38,39,40,41,42,43,44,45,46,47,48,49,50,51,52,53,54], along with 96 additional studies on specific interests (Figure 1). These latter additional studies covered the following topics:

- Predictive microbiology including modeling of microbial growth, inactivation. and survival along the meat chain [55,56,57,58,59,60,61,62,63,64,65,66,67,68,69,70,71,72,73,74,75,76];
- Estimation of the prevalence of contamination at several steps [77,78,79,80,81,82,83,84,85,86,87,88,89,90,91,92,93,94,95,96,97,98,99,100,101,102,103,104,105,106,107];
- Identification of risk factors along the meat chain causing microbial contamination or growth [77,78,80,81,82,83,84,85,87,89,90,91,92,93,97,98,100,101,102,103,104,105,106,107,108,109,110,111,112,113,114,115,116,117,118,119,120,121,122,123,124,125,126,127,128,129,130,131,132,133,134,135,136,137,138,139,140,141,142,143,144,145,146,147];
- Modeling cross-contamination events [74,148,149]; and
- Modeling heat and cooling processes for poultry products [76,150,151].

QMRA studies included in this systematic review were from Europe (23/43), covering France, the Netherlands, Germany, Finland, Sweden, Denmark, Italy, Belgium, the United Kingdom, Sweden, Denmark, Finland, Norway and Iceland); North America (10/43), covering the USA and Canada; South America (2/43), covering Chile and Argentina; Asia (5/43), covering China, South Korea, and Thailand; and one country from Africa (i.e., Senegal) (Table 1).

### 3.2. Poultry Farm-to-Fork Chain

**Figure 2:**
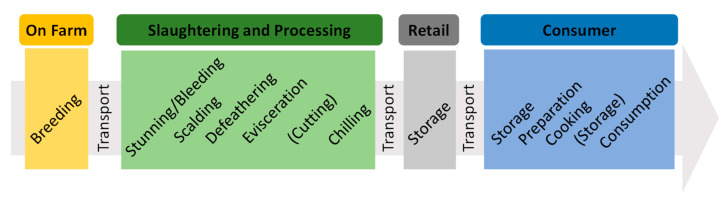
Farm-to-fork chain for poultry meat as commonly considered by quantitative microbial risk assessment (QMRA) studies. Steps within brackets are optional but considered by some authors.

A graphical representation of the farm-to-fork chain for poultry meat products considered by QMRA studies in this review is shown in Figure 2. This was shown to be common to all geographical regions considered in the collected studies. The farm-to-fork chain of poultry consists of some key stages: farm, slaughtering and processing, retail, and consumer. A short description of each step is given below.

#### 3.2.1. Farm

In poultry production, one-day-old chicks are transported from the hatchery to different farms for rearing purposes. This production is accomplished through different farming systems that vary in genotype, housing environments, and rearing methods [8]. In the EU, these systems include extensive indoor, free range, organic, and intensive farming systems [152].

#### 3.2.2. Slaughtering and Processing

Poultry are transported from farms to slaughterhouses. Stages in poultry slaughtering consist of stunning, bleeding, defeathering, evisceration, and processing, which includes the chilling of whole carcasses or further processing like poultry portioning/cutting [8,26,34]. Before transportation to slaughterhouses, chickens are not fed for less than 12 h to minimize the risk of contamination due to fecal leakage during transport [8]. This way, by emptying the bowels, it helps to reduce the skin contamination level by less than 5% after evisceration [8].

#### 3.2.3. Retail

After the processing stage, poultry in different meat cuts or as whole carcasses are transported to retail stores/outlets and kept under 4 °C. In addition, cuts and carcasses can be packaged using specific measures like modified atmosphere or specific packaging materials to ensure better and longer storage of the product.

#### 3.2.4. Consumer

The consumer stage involves the transport of whole chicken carcasses or poultry products from retail store/outlets to home places. Products are either cooked immediately or after storage in refrigerators.

### 3.3. Pathogens Included in the Review of Quantitative Microbial Risk Assessment (QMRA) Studies

**Figure 3:**
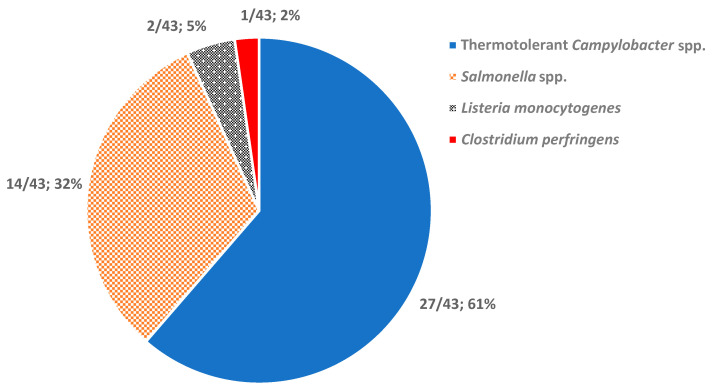
Pathogens included in review of QMRA studies. One paper [51] included QMRAs for both *Salmonella* spp. and thermotolerant *Campylobacter* spp., so it was counted twice.

As illustrated in Figure 3, most of the collected papers dealt with thermotolerant *Campylobacter* spp. (61% 27/43), followed by *Salmonella* spp. (32%, 14/43). Other studies concerned *Listeria monocytogenes* and *Clostridium perfringens* (2/43 and 1/43, respectively).

Regardless of the region, *Salmonella* spp. and thermotolerant *Campylobacter* species were the two first-ranked pathogens in almost all cases [1]. *Salmonella* spp. is acknowledged as the etiologic agent of the human salmonellosis disease while thermotolerant *Campylobacter* species—with *Campylobacter jejuni* ranked first followed by *coli*, *lari*, and *upsaliensis*—are the cause of human campylobacteriosis. Both diseases cause gastroenteritis symptoms (e.g., diarrhea, fever, vomiting, and abdominal cramps) for several days. Nevertheless, salmonellosis can lead to outcomes like sepsis and typhoid fever, in the case of lymphatic system invasion or inflammatory bowel disease. Considering campylobacteriosis, diarrhea can evolve into dysentery while the disease itself can lead to sepsis, but also asthenia, arthritis, endocarditis, or even nerve damage, leading to a Guillain-Barré syndrome outcome.

The two last ranked pathogens were *Listeria monocytogenes* and *Clostridium perfringens*. Listeriosis disease, associated with *Listeria monocytogenes* infection, is a disease known to mainly affect sensitive populations (newborn, pregnant women, immunocompromised, and elderly people) with a very high severity. *Clostridium perfringens* is mostly associated with self-limiting diarrheas caused by thermoresistant enterotoxin A.

### 3.4. Parts of the Farm-to-Fork Chain Considered in QMRA Studies

Steps of the farm-to-fork chain considered in collected QMRA studies are identified in Table 2. 

**Figure 4:**
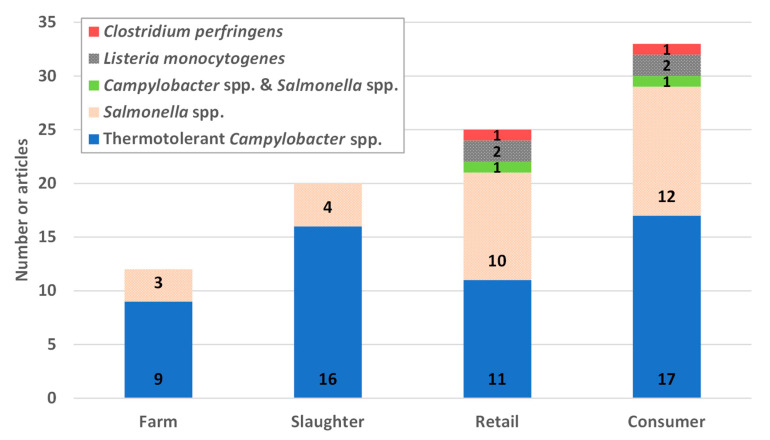
Frequency of farm-to-fork chain steps studied in the QMRA studies.

The overview of collected studies (Figure 4) revealed that most of them focused on *Salmonella spp.* and thermotolerant *Campylobacter* species, especially during the consumption stage.

The consumer part includes four distinct steps: storage, preparation, cooking, and consumption. Many studies have shown that food poisoning outbreaks occur mainly due to improper storage, cross contamination during preparation of meals, and inadequate heating during cooking [153,154,155]. Therefore, these three key aspects were analyzed below.

### 3.5. Focus on the Consumer Step

#### 3.5.1. Effect of Poultry Storage

Poultry products are stored refrigerated or frozen, but in reality, cold chain failures may occur.

Concerning *Salmonella* spp. in poultry, most QMRA studies consider refrigerated storage as an essential step because proper storage conditions limit the growth of bacteria. Thus, most articles considered growth only during temperature abuse events above 5.2 °C, which is considered as the minimal growth temperature for *Salmonella* spp. [13,14,15,17,18,19,20,21], or even no growth at all [23]. For example, a study by Collineau et al. (2020) showed the importance of storage temperature on the risk of salmonellosis [14]. According to this author, the effect of temperature abuse during storage, both at the consumers and in retail, would have a considerable impact on *Salmonella* spp. growth in poultry products. This effect could be more important in retail storage than in consumer storage because of the longer storage duration (average of 3.7 days versus 2.2 days) and the higher temperature achieved (19.4 °C vs. 16 °C). Straver et al. (2007) mentioned that there was little chance of finding a *Salmonella*-free chicken filet after domestic storage, but almost 50% chance to find 0–1 log10 CFU per product [21]. Pouillot et al. (2012) performed a risk assessment on chicken meals prepared in households in Dakar by considering a long storage period at a relatively high ambient temperature before cooking (average time temperature profile: 4 h and 17 min at 29.3 °C) [51]. They showed that this storage before cooking would induce an average growth of 2.1 log10 CFU of *Salmonella* spp. with a maximum of more than 4 log10 CFU for a specific extreme reported time–temperature profile [51].

Additionally, for thermotolerant *Campylobacter* spp., storage conditions (refrigerated or frozen) can have an impact on the level of contamination on poultry carcasses by impairing the growth of bacteria and even reduce contamination levels [37,46,48,71]. The main assumption reported by studies was that thermotolerant *Campylobacter* spp. could not grow below 30 °C, was sensitive to freezing, and could also fairly survive under a cold and humid environment [48,50,156,157,158]. More precisely, Signorini et al. (2013) demonstrated that cold storage prevented thermotolerant *Campylobacter* spp. growth and even led to certain decreased levels, but there was still a proportion of the population that could survive [48,159]. Similarly, Brynestad et al. (2008) [46] reported in Germany a 2-log reduction during freezing with a decrease in viable cells to a very low level on the surface of chicken products. Additionally, Lindqvist and Lindblad (2008) estimated highly variable inactivation rates of thermotolerant *Campylobacter* spp. during the freezing of poultry (from 0.5 up to 3–4 log10 of inactivation) [37,159,160,161,162,163]. However, a data gap remains on temperature along the poultry farm-to-fork chain and more particularly during storage by consumers and also the state in which products are sold (i.e., fresh or frozen) [48]. The risk of human campylobacteriosis was assumed to be 4.10 times less in human when chicken carcasses were kept in frozen storage compared to non-frozen storage [48]. Finally, some studies have estimated the health impacts and found that fresh chicken legs with skin caused more than 90% of campylobacteriosis cases, although they are less consumed than breasts [46].

#### 3.5.2. Impact of Food Preparation

The preparation stage of poultry products is also of concern as risks of cross-contamination are high before cooking [14,16,19,20,51]. During preparation, raw contaminated chicken may be put in contact with other foods or cooked meat ready to be consumed through food contact surfaces including cutting boards or knives, and consequently contaminating them [50]. For instance, Zhu et al. (2017) considered cross contamination in Chinese households via cutting boards as the factor responsible for 92.6% (95% CI, 78.6–99.0%) of the contamination of ready-to-eat (RTE) foods by *Salmonella* spp. [19]. They concluded that on average, 0.51% of consumers might ingest one *Salmonella* spp. cell of each RTE food consumption through transfers from cutting boards contaminated with raw chicken [19]. This is likely to occur, considering that two third of households reported using the same cutting boards for raw chicken and RTE foods and, for half of them, boards were cleaned without any soap or reagents [19]. Consequently, the use of different cutting boards between raw chicken meat and RTE foods was identified as a key strategy to reduce the probability of illness [19,20,51], while an increased serving size was identified as a driver for an increase in *Salmonella* spp. [25]. The prevention of cross-contamination could correspond to a 28% decrease in the average likelihood of illness [14].

Concerning cross contamination of thermotolerant *Campylobacter* spp., food mishandling appears as the main factor of human campylobacteriosis. According to Brynestad et al. (2008), 74% of campylobacteriosis cases in Germany were caused by cross contamination due to unhygienic practices such as not washing hands after handling raw chicken meat and no proper cleaning of kitchen utensils, resulting in 39% and 35% of campylobacteriosis cases, respectively [46]. Nevertheless, available data are very scarce concerning consumer behavior in the kitchen as identified by Uyttendaele et al. (2006) for the Belgian population [50]. According to their observations, between 25 to 76% of consumers in Belgium mishandle raw poultry at home [50]. In this study, it was noted that during cross contamination events, only bacteria present at the surface of meat were transferred [50]. Lindqvist and Lindblad (2008) estimated transfer rates during cross-contamination events from chicken to RTE foods or hands as within a range of 0.02% to 10% [37]. Concerning the impact of the contaminated surface, a study by Calistri and Giovanini (2008) showed that *Campylobacter jejuni* cells transferred from hands and utensils to RTE foods were linked to campylobacteriosis incidence in two Italian regions [47]. On the other hand, the QMRA model by Rosenquist et al. (2003) identified that cross contamination via unwashed cutting boards was supposed to be the most important route of transfer [39]. Signorini et al. (2013) estimated that thermotolerant *Campylobacter* spp. doses ingested by consuming contaminated salad due to mishandled poultry meat products in Argentina was of one or two bacteria per serving in most cases and the contamination prevalence was estimated around 33% [48]. This paper also detailed the impact of hygienic practices adopted in private kitchens on the number of human campylobacteriosis cases. It articulated that adopting good hygienic practices in the kitchen could lower the exposure to thermotolerant *Campylobacter* spp. and hence lower the number of cases [48]. According to estimations made by these authors, if the cutting boards were washed instantly after handling raw poultry products, then the risk of human campylobacteriosis would undergo a ten-fold decrease approximately in comparison with the absence of cleaning [48]. In contrast, using the same unwashed cutting board to prepare the salad would increase the chances of salad contamination in comparison with the use of a washed cutting board [48]. The risk of human campylobacteriosis was also impacted by the preparation sequence If the same cutting board were used to prepare the whole meal, where RTE foods like salad should be prepared before raw poultry meat products, as if uncooked/raw chicken is prepared before salad, there is higher risk of causing campylobacteriosis than following the recommended sequence [48]. Authors also mentioned hand washing as an important step during food preparation at home with a risk increase by >1.4 if hands were unwashed [48]. In its 2018 report, Anses tested hand washing and utensil cleaning interventions in the kitchen as well as the combination of both [8]. In the case of full compliance, the risk reduction achieved is estimated to be 1%, 85%, and 87% for hand washing, utensil cleaning, and combination of both, respectively.

#### 3.5.3. Inactivation of Bacteria during Cooking

During poultry cooking, pathogens can be inactivated by heat treatment. However, some pathogens or their toxins may survive and cause illness after meal consumption. Depending on the cooking method, inactivation is more likely achieved on the product surface rather than on the core, as for pan cooking.

For *Salmonella* spp. in broilers, most authors considered that undercooking was an important factor of risk increase, emphasizing the importance of heat treatment [14,15,16,18,20,22,23,104]. As an indication of *Salmonella* spp. thermoresistance for chicken cooking, the D-value at 70 °C was around 6 s and 24 min at 55 °C, considering a *z*-value of 6.262 °C [164]. Thermal inactivation depends on cooking temperature/time parameters, product shape and size as well as cooking method, but also the serotype and physiological state of cells [16]. Considering cooking of a whole poultry in oven, for example, non-uniform temperature distribution may result in “low-heat” zones protecting cells from inactivation [14,165]. According to the *Salmonella* spp. D value, a minimum cooking process is necessary, for a non-frozen chicken, with a core temperature of the product of 60 °C, for at least 8 min to reduce *Salmonella* spp. to a level at which the risk can be considered as negligible [164]. Achieving a high core temperature is likely to be easier for smaller products in combination with a suitable cooking method and may help to reduce illness risks. For example, a study by Bemrah et al. (2003) indicated that the risk of salmonellosis in French ‘Cordon bleu’ (reconstituted turkey meat turnover coated with breadcrumbs) was close to zero if the cooking temperature achieved at least 63 °C at the core during oven cooking [22]. If the food was cooked using a fryer (i.e., shorter cooking time at a mean core temperature of 57 °C), the risk could not be eliminated, especially with a high initial number of bacteria [22]. Concerning the impact of cooking methods, stir-frying associated with traditional Korean and Japanese recipes like chicken sashimi does not enable the product to reach an internal cooking temperature as high as in frying and boiling and may lead to undercooked chicken meat products. This can sometimes also favor cross-contamination of vegetables from meat [23]. As an example, Oscar et al. (1998) considered in their modeling approach of the cooking step that 20% of chicken were undercooked, which could induce 1% to 10% survival of the *Salmonella* spp. population [15]. Cooking chicken to a mean temperature of 62 °C (min = 55 °C; max = 70 °C) for a mean time of 30 min (min = 15 min; max = 45 min, was estimated to decrease the number of contaminated chickens from 3000 to 16, with a mean *Salmonella* spp. level of 4.7 cells per contaminated chicken (min: 1 cell; max: 21 cell) [16]. Thus, the cooking temperature and microbial concentration had a predominant effect on the resulting probability of illness [16,24] and adequate cooking would be even more important than the prevention of cross contamination with a reduction of salmonellosis risk by 64.3% and 27,6%, respectively [14]. In addition, cooking unthawed frozen ground turkey burger observed in 2.2% of cases, resulted in 38–52% of the salmonellosis case numbers at home [24].

Concerning *Campylobacter,* Uyttendaele et al. (2006) described that under adequate heat treatment of poultry meat, thermotolerant *Campylobacter* spp. barely survives in poultry products due to the heat sensitive properties of this pathogen [36,50]. It concluded that improper heating of chicken products might result in higher exposure probability to the pathogen. This was reported by Brynestad et al. (2008), who stated that undercooking of a poultry product contaminated by thermotolerant *Campylobacter* spp. caused 3% of German illness cases [46]. During undercooking, up to 20% of thermotolerant *Campylobacter* spp. cells are able to survive in protected areas of the poultry product [40,41,50]. This proportion of cells was determined by the report of the FAO/WHO (2002), describing that once the outside heating temperature of a meat product had reached 74 °C, then the inside temperature was around 60–65 °C within 0.5 to 1.5 min [40,41,165]. This paper also mentioned that since the D-value of thermotolerant *Campylobacter* spp, for poultry was less than 1 min at 60 °C, compared to 3.8 min for *Salmonella* spp. [164], it should be efficiently inactivated by temperatures set to inactivate *Salmonella* spp. [166].

#### 3.5.4. Influence of the Consumer Behavior during Serving

Consumer behavior during serving refers to at-home practices after product cooking that may have an impact on risk of illness. Regarding *Salmonella* spp., potential storage conditions after cooking, duration before consumption, and dressing with utensils previously used for raw meat were investigated [16,17,20,21,22,51,104,114]. Additionally, a *Salmonella*-free meal could be contaminated from the use of the same cutting boards, utensils, or unwashed hands as for the raw meat preparation for cooked meal dressing or salad preparation (see Section 3.5.4 for the latter) [20,104,114]. For instance, a Canadian survey estimated that 0.6% of consumers did not wash their hands and 1%, their cutting boards after raw meat handling [20]. By aggregating data from several studies, they estimated the transfer from contaminated hands varying between 0.14% and 52.95% with a most likely value of 8.93% and the transfer from boards varying between 10.49% and 42.38%, with a most likely value of 19.40% [20]. Oscar estimated that this pathogen transfer could nearly double the contamination level of cooked chicken at consumption from 21 to 39 cells [16], and later identified a linear relationship between frequency of improper serving and salmonellosis incidence [17].

Concerning thermotolerant *Campylobacter* spp. in poultry meat preparations, Uyttendaele et al. (2006) described that in order to reduce exposure, limiting the consumption of raw/uncooked meat is also important, along with reducing the contamination level of thermotolerant *Campylobacter* spp. Consequently, communication campaigns are required to warn consumers of the risks in consuming raw poultry [50]. Concerning cross-contamination of cooked chicken, *Campylobacter* species are known to be able to easily transfer (i.e., in 80% of contact events of 10 s from raw meat to cutting board and in 30% of contact events from the board to the cooked meat) [8]. This kind of direct contamination from utensils/hands has also been considered in several other studies [39,45,46,47,48,50]. Cross-contamination when using unwashed utensils/hands was also simulated by developing a “drip-fluid” model. This model considered the leakage of a contaminated fluid, consisting of a mixture of chicken blood and residual water gained during processing and during meal preparation [30,40,41]. Nevertheless, data gaps persist for some factors like the impact of contact area between raw meat and board as well as the time elapsed between consecutive contacts of raw followed by cooked meat with the board [39].

## 4. Discussion

### 4.1. Summary of Mitigation Interventions Applicable at the Consumer Step

Considering the 43 QMRA studies collected, most studies addressed the impact of the consumer behavior. The main risk factors and mitigation measures in the consumption step were identified in Table 3 for the main pathogens *Salmonella* spp. and thermotolerant *Campylobacter* spp. Consumers have an impact on the final level of contamination of the product due to conditions of storage, preparation, cooking, and post-cooking handling. In particular, cross-contamination occurring during both food preparation and final dressing as well as undercooking of the meat were put forward as high risk factors to address. To avoid pathogen survival and growth, measures suggested to be taken by consumers include thawing frozen meat before cooking, use of a meat thermometer to ensure the target internal temperature, prepare side ingredients before the raw meat, or never reuse an unwashed cutting board for both raw and cooked meat. Cooking can inactivate *Salmonella* spp. at 70 °C for at least 1 min and thermotolerant *Campylobacter* spp. at temperatures above 60 °C for longer than 1 min.

### 4.2. Geographical Specificities within the Poultry Meat Chain

It has to be noted that regardless the country, *Salmonella* spp. and *Campylobacter* spp. are very often ranked amongst pathogens most associated with the burden of foodborne diseases [1]. *Salmonella* spp. and *Campylobacter* spp. were also found as the top microbial hazards considered in the risk assessment of the poultry chain regardless of country [23,25,28,48,51]. Regarding the guidelines and regulations, these were generally found to be similar, especially in developed countries. However, some specificities associated with regions, customs, or individual behaviors were also observed [8,28,51,54,167].

Concerning processing, there were almost no differences considering the steps occurring at the slaughterhouse [9], except for some specificities related to carcass decontamination, as underlined by the EFSA and Anses reports [8,28]. Thus, chemical decontamination of poultry carcasses, which encompasses washes using organic acids, chlorinated, or electrolyzed water as well as peracetic acid, acidified sodium chlorite, or trisodium phosphate is currently not authorized for any poultry products intended to be sold in the EU market, contrary to other non-EU regions [28]. Moreover, several EU countries prohibit the use of irradiation, either gamma radiation or x-rays, for carcass decontamination [28]. There are also some mitigation strategies that are routinely applied only in some countries (Norway, Iceland, and Denmark) like scheduled slaughtering, which consists in the testing of carcasses before slaughter to identify positive carcasses requiring additional microbial reduction treatments compared to negative ones [28].

Considering the other parts of the meat chain, more differences may occur at the farm, retail, and at the consumer points. If no data were available in the collected papers concerning the farm step, two papers provided information concerning the two latter steps in developing and East Asian countries [23,51]. Thus, considering retail in Senegal, the dedicated “market-to-fork” published by Pouillot et al. pointed out that chickens were sold alive and slaughtered at the time of sale, without prior refrigerated storage [51]. This greatly differs from what is known about cold chain compliance regulations concerning the retail sale of poultry meat. On the other hand, Jeong et al. explicitly considered South Korean cooking behavior observed for traditional dishes to build its QMRA study, which implies an increased risk of meat undercooking [23]. Moreover, authors have highlighted local cooking specificities that can have a significant impact on food safety, with the examples of chicken stir-frying and sashimi (meat consumed raw).

Among all steps of the poultry farm-to-fork chain, the consumer’s behavior represents the most variable factor across geographical regions because it is related to cultural preparation and cooking habits [8,167]. This is pushed forward by the FAO and WHO, who consider that little is known about the consumer’s behavior and that its monitoring remains difficult, despite being reliable [168]. Thus, a great discrepancy of behaviors can be observed at the global scale as well as at individual scale. At a global scale, several specificities are observed according to regions. When considering African regions, Pouillot et al. emphasized the fact that consumers tend to expose chicken carcasses to hazardous environmental conditions (i.e., storage at temperature above 17.5 °C for several hours) [51]. Other examples can be given when considering consumers from developed countries, with specificities associated with traditional recipes leading to potential undercooking [23] or general consumer compliance to hygienic practices associated with ethnicity, gender, or education [8,167].

### 4.3. Consumer Education with Regard to the Whole Farm-to-Fork Chain

Authorities and managers also have to make sure that mitigation measures are applied along all parts of the farm-to-fork chain and more particularly, interventions realized at the consumer step as they are expected to have the biggest impact on the risk reduction of illness. Indeed, upstream efforts at any stage that prevent contamination and reduce the contamination level will later reduce illness risk to a greater extent.

The impact of mitigation interventions applied at the pre-slaughter, processing, and post-processing stages was assessed by authors by testing alternative scenarios and conducting sensitivity analysis. Among the QMRAs collected, some studies covered the whole farm-to-fork chain and assessed interventions and risk factors for every part of it [8,14,18,28,30,32,33,34]. The majority of studies agree on the high impact of the role of the consumer on the microbial safety of the serving [8,14,30]. For instance, risk reductions could be decreasingly achieved by adequate cooking, avoiding cross-contamination by utensils and hand washing, and compliance with adequate storage temperature [14,30]. In contrast, two studies considered that the intervention measures most efficient in risk reduction were rather found in the processing and pre-processing steps by limiting fecal leakage during processing, scheduled slaughtering, and decontaminating flocks [32,34].

Apart from the consumer behavior and considering both pathogens of interest, efforts should focus on reducing the introduction of contaminated animals into the slaughterhouse by intervening at the farm [8,14,32,34], avoiding cross-contamination at the slaughterhouse [14,30,32], and ensuring proper cold storage at retail points [14,34]. More precisely, farm interventions such as cleaning between flocks are expected to reduce illness risk by 16% [8], but measures like poultry vaccination of phage usage, which are currently unavailable, may reduce it up to 93% [8,32,34]. Increased caution to reduce contamination spreading at scalding, evisceration, plucking, and even avoiding chilled baths can achieve high risk reductions [8,30,32].

When assessed, combined interventions always turn out to be the most efficient, especially when covering the whole farm-to-fork chain [8,30]. As an example, by combining the most efficient interventions at each stage of the chain, it is possible to reduce the campylobacteriosis risk by 99% [8]. Risk management through guidelines and regulations aim to target risk reduction throughout the meat chain. However, risk management at the level of the consumer remains difficult. It may only be achieved by consumer education on adequate handling and cooking practices, which, if thoroughly applied by the population, could have a greater impact on public health compared to other risk reduction methods [169,170,171]. In parallel to these communication campaigns, data gaps need to be addressed when speaking of consumption habits. Large uncertainty remains concerning the latter, impairing both robustness and efficiency of QMRAs and the design of education campaigns. To do so, national-level surveys are regularly conducted by several countries, revealing relevant information on, for example, the duration and mean refrigeration temperature of the storage of meat after purchase or the willingness to wash hands and utensils between preparations [8,16,19,20,24,39,40,46,48,50]. When not available or not suited to their studies, authors had to conduct such surveys by themselves [19,51]. In a guidance for microbiological risk assessment of food, the FAO and WHO have also suggested gathering data as quantitative and descriptive as possible regarding temperatures, durations, level of contamination, and transfer rates to be able to use predictive models to assess the level of contamination at these specific steps. Observation of food handling practices through purpose-built food preparation kitchens and video captures are also encouraged [168]. Quantitative data are indeed crucial for QMRAs to assess the impact of consumer habits on the risk of illness and evaluate and rank the efficiency of intervention measures [24].

Additionally, these surveys help to adapt communication campaigns to different cooking habits affected by factors like culture. Among the collected papers, some dealt with the cooking habits of African and Asian cultures, with their specificities and limitations [23,51]. Pouillot et al., while assessing salmonellosis and campylobacteriosis risks from consumption of home-prepared meals in Dakar (Senegal), highlighted the absence of refrigerated storage and the difficulties to achieve proper pathogen removal during slaughter (done at market) [51]. Jeong et al. emphasized the impact of traditional cooking methods (i.e., cooking in a pan or fryer) that fail to achieve effective heat treatment at the product’s core [23]. Moreover, not considering these traditional methods can lead to voluntary ignorance or rejection of education campaigns, as observed in other circumstances such as medical burials during the last Ebola outbreak [172]. Education campaigns should also be presented as advice rather than regulations to be followed in order to reach a greater audience. Several methods can be used such as the nudge method. Nudges can be used along with education to incite the consumer to follow guidelines by making it easy and beneficial for them [173]. Finally, frequent surveys help to estimate the impact of education campaigns on the improvement of handling behaviors in kitchens [169,170,171].

## 5. Conclusions

Poultry contamination by pathogenic bacteria is known to occur at almost every step of the farm-to-fork chain. The use of a risk-based assessment method to improve general food safety is a key strategy currently promoted by national and international health authorities in all food sectors including poultry. However, the amount and heterogeneity of QMRA studies available make it difficult to easily grasp all the risk factors, data gaps, and areas for improvement. To bring some light on the most relevant issues to address, this review summarized available data from published QMRA studies dealing with poultry meat contamination using a systematic review procedure.

This study collated the available scientific resources regarding quantitative microbial risk assessment studies in poultry meat. In doing so, it highlights the effective implementation of the PRISMA methodology in using evidence-based data sources to inform an evaluation of intervention methods, resulting in an emphasis in this study on the central role of consumer behavior in influencing food safety risk. Consumers have a key role to play for the end-product safety. This is especially true regarding the new consumption trends appearing today such as the rise of homemade meals or storage habits, and the fact that consumer food handling is the final step of the meat chain. Many uncertainties remain concerning the consumption habits of the population, varying with social position, location, and cultural aspects. However, by characterizing the specific features associated with those trends and aspects and thus improving knowledge of the population, QMRAs can be fed with high quality data, leading to more accurate risk assessment as well as intervention strategies and education campaigns. Thus, ascertaining more data regarding consumer habits should lead to improved understanding of pathogen interactions in the domestic environment with the ultimate aim to reduce their persistence and improve public health. This study provided an expert overview of the importance of the role of the consumer and the issues associated with the variability of storage and cooking behaviors as well as the difficulty to assess these behaviors.

## Figures and Tables

**Table 1:** Summary of collected poultry meat QMRA papers.

Pathogen	Product	Location Date	Population	Objective	Ref
*Salmonella spp.*	Broiler	Canada 2012	All	Meta-analysis for evaluation of farm-to-processing interventions	[13]
		Canada 2020	All	Risk assessment, evaluation of mitigation strategies	[14]
		Finland 2005	All	Effect of two interventions of Finnish control program	[18]
		China 2017	All	Risk assessment of home-prepared chicken meals	[19]
	Chicken	USA 1998	All	Determination of *Salmonella* spp. contamination levels on chickens at processing plant exit and risks for consumers	[15]
		USA 2004	All	Risk assessment based on updated model from [15]	[16]
	Chicken meat	USA 2017	All	Risk assessment following flow pack wrapping of whole chicken and temperature abuse	[17]
		Canada 2013	All	Risk assessment of home-prepared chicken meals	[20]
		The Netherlands 2007	All	Risk assessment, impact of contamination level at retail	[21]
	Meat preparation ^1^	France 2003	All	Risk assessment at catering establishments level	[22]
		South Korea 2018	All	Risk assessment, impact of contamination concentration at retail	[23]
		USA 2019	All	Risk assessment for developing a risk management framework	[24]
		USA 2019	All	Process risk model for ground chicken, partly based on [17]	[25]
Thermotolerant *Campylobacter* spp.	Broiler	UK 2001	All	Risk assessment for broilers at point of slaughter	[26]
		UK 2017	All	Assessment of mitigation interventions at farm level	[27]
		EU 2011	All	Impact of mitigation interventions at primary production and slaughter	[28]
		EU 2013	All	Impact of farm to fork interventions on human campylobacteriosis incidence	[29]
		USA 2019	All	Assessment of processing interventions	[30]
		The Netherlands 2005	All	Risk assessment, poultry processing model basis	[31]
		Sweden 2008	All	Evaluation of mitigation strategies and frequency of cross contamination due to consumer mishandling	[37]
		United Nations 2009	All	Risk assessment based on extensive review of knowledge	[40,41]
		Nordic countries 2013	Young, adult, males	Establishment of risk-based microbiological criteria	[42]
		France 2018	All	Risk - benefit assessment of mitigation interventions	[8]
	Chicken	Denmark 2003	All	Risk assessment, impact of mitigation strategies	[39]
		China 2013	All	Prevalence estimation and risk assessment	[36]
	Poultry	Thailand 2011	All	Exposure assessment and processing risk factors identification	[38]
		China 2018	All	Risk assessment based on poultry-processing model	[35]
	Chicken meat	Denmark 2012	All	Evaluation of control strategy for imported, meat	[43]
		Denmark 2013	All	Risks associated with thermotolerant *Campylobacter*, based on [49]	[44]
		Denmark 2013	All	Risk assessment for individual batches of fresh poultry meat	[45]
		Netherland 2007	All	Tool for mitigation measures assessment	[32,33,34]
		Germany 2008	All	Risk assessment of frozen/fresh chicken legs and breasts, for household consumption	[46]
		Italy 2008	All	Risk assessment of human campylobacteriosis due to cross contamination	[47]
		Argentina 2013	All	Risk assessment for cross-contaminated salad	[48]
		EU 2012	All	Impact of microbial criteria at the end of industrial processing	[49]
	Meat preparation ^1^	Belgium 2006	All	Support for definition of risk-based microbial criteria	[50]
*Campylobacter* spp. and *Salmonella* spp.	Chicken meat	Senegal 2012	All	Risk assessment considering from market to consumption	[51]
*Listeria monocytogenes*	Poultry	Chile 2015	All	Risk assessment for both poultry and beef meat	[52]
	Broiler legs	Finland 2008	All	Plant-level risk assessment	[53]
*Clostridium perfringens*	Meat preparation ^1^	USA 2009	All	Effect of maximum allowed growth during stabilization of ready-to-eat foods	[54]

^1^: “Meat preparation” referred to portioned, cut, or minced meat to which spices or other ingredients might have also been added to improve sensorial properties or texture. Sausages and hamburgers of raw minced poultry meat were included as meat preparation products.

**Table 2:** Identification of steps of the farm-to-fork chain considered in poultry QMRA papers.

Pathogen	Reference	Farm (12)	Slaughter (20)	Retail (25)	Consumer (33)
*Salmonella* spp.	Bucher et al. 2012 [13]	✓	✓		
	Collineau et al. 2020 [14]	✓	✓	✓	✓
	Oscar 1998 [15]		✓	✓	✓
	Oscar 2004 [16]			✓	✓
	Oscar 2017 [17]			✓	✓
	Maijala et al. 2005 [18]	✓	✓	✓	✓
	Zhu et al. 2017 [19]			✓	✓
	Smadi & Sargeant 2013 [20]			✓	✓
	Straver et al. 2007 [21]			✓	✓
	Bemrah et al. 2003 [22]			✓	✓
	Jeong et al. 2018 [23]			✓	✓
	Sampedro et al. 2019 [24]				✓
	Oscar et al. 2019 [25]				✓
Thermotolerant *Campylobacter* spp.	Hartnett et al. 2001 [26]	✓	✓		
	Crotta et al. 2017 [27]	✓	✓		
	EFSA 2011 [28]	✓	✓	✓	✓
	Romero-Barrios et al. 2013 [29]	✓	✓		✓
	Dogan et al. 2019 [30]	✓	✓	✓	✓
	Nauta et al. 2005 [31]		✓		
	Katsma et al.; Havelaar et al.; Nauta et al. 2007 [32,33,34]	✓	✓	✓	✓
	Huang et al. 2018 [35]		✓		
	Lindqvist and Lindblad 2008 [37]		✓	✓	✓
	Osiriphun et al. 2011 [38]		✓		
	Rosenquist et al. 2003 [39]		✓	✓	✓
	Wang, Guo & Li, 2013 [36]	✓	✓		✓
	FAO, WHO 2009 [40,41]	✓	✓		✓
	Nauta et al. 2013 [42]				✓
	Anses 2018 [8]	✓	✓	✓	✓
	Boysen 2012 [43]		✓	✓	
	Boysen et al. 2013 [44]				✓
	Christensen et al. 2013 [45]			✓	✓
	Brynestad et al. 2008 [46]			✓	✓
	Calistri & Giovanini 2008 [47]				✓
	Signorini et al. 2013 [48]		✓	✓	✓
	Nauta et al. 2012 [49]				✓
	Uyttendaele et al. 2006 [50]			✓	✓
*Salmonella* spp. and *Campylobacter* spp.	Pouillot et al. 2012 [51]			✓	✓
*Listeria monocytogenes*	Foerster et al. (2015) [52]			✓	✓
	Aarnisalo et al. (2008) [53]			✓	✓
*Clostridium perfringens*	Golden et al. (2009) [54]			✓	✓

**Table 3:** Main risk factors and mitigation measures suggested in the consumer step.

Step	Risk Factors	Risk Mitigation Measure	References
Storage	Temperature abuses	Respect of temperatures <4 °C	[14,16,17,19,21,48,50,51]
		Monitoring	[48]
	Survival of thermotolerant *Campylobacter* spp. in fridge	Freeze (−20 °C, ≥24 h)	[32,37,43,44,46,48]
	Cross-contamination	Change/wash utensils and wash hands between preparations	[8,19,20,46,48,50,51,166]
		Consumer education	[14,32]
		Prepare raw meat after other ingredients	[48]
Cooking	Undercooking	Thaw frozen meat before cooking	[24]
		Adapt cooking methods to product’s shape and size to facilitate heat transfer	[16,22,25]
		Core temperature >70 °C	[14,16,18,20,22,23,24,25,40,41,46,51]
Consumer behavior	Storage after cooking	Store at heat (stove for example)	[22]
	Cross-contamination	Change or wash utensils and hands between preparations	[8,16,17,20,30,32,39,40,41,45,46,47,48,50,51,166]
		Consumer’s education	[14]
	Consumption of raw meat	Communication campaigns to limit	[23,32,50]
	Data gaps	National surveys on consuming behaviors	[39]
